# Understanding the spatio-temporal dynamics of meningitis epidemics outside the belt: the case of the Democratic Republic of Congo (DRC)

**DOI:** 10.1186/s12879-020-04996-7

**Published:** 2020-04-20

**Authors:** Serge Mazamay, Didier Bompangue, Jean-François Guégan, Jean-Jacques Muyembe, Francis Raoul, Hélène Broutin

**Affiliations:** 1 Département de Microbiologie, Faculté de Médecine, Université de Kinshasa, Kinshasa, Democratic Republic of Congo; 2 MIVEGEC, UMR IRD CNRS UM, 911 avenue Agropolis, BP 64501, 34394 Montpellier Cedex 5, France; 3 UMR CNRS 6249 Chrono-Environnement, Besançon, France; 4 ASTRE UMR INRAE Cirad UM, Campus International de Baillarguet, 34398 Montpellier 722 Cedex 5, France; 5 Département de Parasitologie-Mycologie, Faculté de Médecine, Université Cheikh Anta Diop (UCAD), Dakar, Senegal; 6 CREES (Centre de Recherche en Ecologie et Evolution de la Santé), Montpellier, France

**Keywords:** Epidemics, Eco-epidemiology, Spatial clusters, Seasonality

## Abstract

**Background:**

Bacterial meningitis remains a major threat for the population of the meningitis belt. Between 2004 and 2009, in the countries of this belt, more than 200,000 people were infected with a 10% mortality rate. However, for almost 20 years, important meningitis epidemics are also reported outside this belt. Research is still very poorly developed in this part of the word like in the Democratic Republic of Congo (DRC), which experiences recurrent epidemics. This article describes for the first time the spatio-temporal patterns of meningitis cases and epidemics in DRC, in order to provide new insights for surveillance and control measures.

**Methods:**

Based on weekly suspected cases of meningitis (2000–2012), we used time-series analyses to explore the spatio-temporal dynamics of the disease. We also used both geographic information systems and geostatistics to identify spatial clusters of cases. Both using conventional statistics and the Cleveland’s algorithm for decomposition into general trend, seasonal and residuals, we searched for the existence of seasonality.

**Results:**

We observed a low rate of biological confirmation of cases (11%) using soluble antigens search, culture and PCR. The main strains found are *Streptococcus pneumoniae*, *Haemophilus influenzae* and *Neisseria meningitidis* (A and C) serogroups. We identified 8 distinct spatial clusters, located in the northeastern and southeastern part of DRC, and in the capital city province, Kinshasa. A low seasonal trend was observed with higher incidence and attack rate of meningitis during the dry season, with a high heterogeneity in seasonal patterns occurring across the different districts and regions of DRC.

**Conclusion:**

Despite challenges related to completeness of data reporting, meningitis dynamics shows weak seasonality in DRC. This tends to suggest that climatic, environmental factors might be less preponderant in shaping seasonal patterns in central Africa. The characterization of 8 distinct clusters of meningitis could be used for a better sentinel meningitis surveillance and optimization of vaccine strategy in DRC. Improving biological monitoring of suspected cases should be a priority for future eco-epidemiological studies to better understand the emergence and spread of meningitis pathogens, and the potential ecological, environmental drivers of this disease.

## Backgrounds

Global burden of bacterial meningitis is estimated to 5 million of new cases and 290,000 deaths globally from meningitis in 2017 [1], mainly due to three major bacteria: *Neisseria meningitidis* (Nm), *Streptococcus pneumoniae* (Sp) and *Haemophilus influenzae type b* (Hib) [2]*.* Bacterial meningitis remains a major public health in Africa and in particular in the Sub-Saharan region stretching from Senegal to Ethiopia, called the « Meningitis Belt » [3–6].

In this highly infected region, seasonality of epidemics is clearly described with peaks during the dry season. Climatic parameters are more and more described as main factor of the epidemics pattern, including aerosols, Harmattan winds and humidity [7–10]. Very low humidity and dust can stimulate meningococcal invasion by directly damaging the mucosal barrier or by inhibiting immune defenses of the upper respiratory tract surface in humans [11]. The epidemiology and the dynamics of bacterial meningitis is largely described and now better understood in the African meningitis Belt [9].

In contrast, the dynamics of bacterial meningitis outside the belt in Africa is still poorly explored despite epidemics occurring regularly such as in Uganda [12], in Kenya [13, 14], in Southwest Cameroon and in the Democratic Republic of Congo (DRC) [15, 16], which reports cases annually to the World Health Organization (WHO) since 1937 [3, 15].

Sporadic cases of meningitis have been reported previously in different districts of DRC for more than 20 years: Aru in 1937, Mahagi in 1937, Gemena in 1960, Kalemie in 1960, Rutshuru in 1960. The main strain found was Nm A [3]. The resurgence of meningitis epidemics in these former sporadic case reporting areas, such as Aru in 1996 with 86 cases including 11 deaths and Inongo (former Bandundu Province) in 2002 caused by the Nm A [17], raises interest in understanding the epidemiology of this disease outside the known endemic Sahelo-Sudanian belt [18]. Meningitis outbreaks are observed outside the meningitis belt while the determinants of meningitis dynamics in this part of Africa remain unexplored. These epidemics are behind the hypothesis of the emergence of a southern meningitis belt as illustrated in few studies [4, 19, 20].

In this study, based on a 13-year time-series of suspected meningitis cases reported in all 515 health zones in DRC, we provide a first spatio-temporal analysis of meningitis for this country. The purpose of this analysis is to describe the seasonal and medium-term dynamics of meningitis outside the African meningitis belt, in a region of a predominantly forested environment which is highly distinct from the classic meningitis belt.

## Methods

### Study area

The DRC is an African country crossed by the Equator, located between the 5° North latitude and 13° South latitude, with a 2,345,409 km2 surface area and that shares limits with 9 countries. The population size is estimated at 65.8 million inhabitants with about 50% of under 15 years old, and 55–60% of the total population living in rural areas.

DRC presents diverse reliefs including a basin in the center of the country (48% of the total surface), with a mean altitude of 350 m and covered by dense tropical forests. Uplands surround this part of the country up to the borders except in the east part where uplands end with mountains with a mean altitude higher than 1000 m. This geographic mosaic also induces 4 different climates across the country: an equatorial climate in the center of the country (basin), a tropical and humid climate in North and South, a temperate climate in the east and a mountain climate in the extreme east [21].

Additionally, to the dense tropical forest in the basin, the important river network (i.e., the Congo river of 4700 km and its numerous tributaries and lakes), constitutes an important geographic characteristic of the country. The socio-economic situation of the population is very poor, especially in rural areas where populations live from agriculture, fishing and hunting.

The health system is organized into 3 administrative levels, central, intermediary and peripheric. Until to 2015 (i.e., including the study period), the country was divided into 11 sanitary provinces, 65 sanitary districts, 515 health zones (a geographical entity of an average of 6000 to 10,000 km2 including a population of at least 100,000 inhabitants) and 8504 health areas (a geographical entity of an average of 300 to 500 km2 including a population of at least 10,000 inhabitants) [22].

### Epidemiological data

We collected epidemiological data from 3 different sources:

- Passive surveillance

We collected the weekly numbers of suspected cases reported from Integrated Diseases Surveillance System (IDSR) implanted by the national health system from the Ministry of Health, between 2000 and 2012, at the health area spatial scale. Suspected cases were defined according to the WHO guidelines: “any person with a high fever of sudden onset (rectal temperature > 38.5°C or axillary > 38°C) and one of the following signs: stiffness of the neck, changes in consciousness or other meningeal signs”. In patients under 1-year of age, a suspected case of meningitis occurs when fever is accompanied by a bulging fontanel [23].

- Active surveillance

Data were collected from sentinel health zone sites, attached to health areas in the former province of Katanga (Lubumbashi), from the city-province of Kinshasa (Lingwala, Kalembelembe and Kingasani) and others provinces (Bandundu, Bas-Congo, Equateur, Kasai Oriental, Kasai Occidental, Maniema, Province Orientale, Nord Kivu and Sud Kivu).

The dataset includes suspected cases identified (see Additional file 1) and for which a cerebrospinal fluid (CSF) sample was taken by the health staff of these structures (sentinel sites), other reference centers and in the referral hospitals.

Patients with suspected meningitis who were evaluated at reference structures or at sentinel sites structures had a lumbar puncture performed to collect CSF for laboratory testing by Gram stain and culture or latex agglutination, where these were available or Polymerase Chain Reaction (PCR) which is available since 2012 at the Institut National de Recherche Biomédicale (INRB) in DRC.

Laboratory methods for culture:

Seeding on culture media, Isolation, reading and interpretation, serotyping, antibiogram, preservation of isolated strains. The method used is Fresh Blood Agar Culture (GS), Blood cooked and Enriched chocolate agar/VCN, incubation at 37 °C under CO2 for 24 h.

Laboratory methods for PCR:

DNA Extraction, Amplification / Elongation, Hybridization is used. Kits exist for the specific research of certain germs such as meningococcus, pneumococcus and Hib, rt. PCR for Enterovirus, PCR Species / Nm (A, B, C,W,X,Y), PCR Species / Hi b/Hi a and PCR Species / SP

- Case reports

We collected data and contextual information from reports on meningitis epidemic investigations performed by the Ministry of Health and different health non-governmental organizations which intervene in the epidemic response (Médecins sans Frontières, Epicentre and WHO).

It is important to note that active surveillance data, passive surveillance data and case reports are integrated into the Integrated Diseases Surveillance System (IDSR) global database of the Ministry of Health, DRC. All these data were cross-referenced to those of the case confirmation database acquired at the INRB in order to ensure laboratory results (identified strains) and to highlight the context of epidemics in the country.

### Laboratory data (confirmed cases)

We obtained the database of confirmed cases from 2008 to 2012 in DRC from the INRB which presents the information about all CSF samples received for the meningitis diagnostic, including the date of sampling, the pathogen identified by culture or PCR.

### Population data

Population sizes of health areas were collected from the Expanded Programme on Immunization (EPI), program of Public Health Ministry (Programme Elargi de Vaccination - PEV). They correspond to estimations based on census performed in 1984 with a calculated annual growth rate of 1.03% applied for the following years.

### Shapefile data

Issued from WHO Healthmapper® (a WHO information and mapping application for public health).

### Data analysis

Analyses were performed with softwares QGIS® 1.8.0 (QGIS project 1.8.0, 2013), SaTScan® v9.1.1 (Kulldorff and Nagarwalla, 1995) [24–26], Stata® ver. 16 (StataCorp. LLC, 2019) and *R*® (3.0.1 *R* version, 2013).

- Development of thematic maps: Maps showing average attack rates (2000 to 2012) by province and health zones (per 100,000 inhabitants) were performed using QGIS, using base maps extracted from Healthmapper® software produced by WHO. Health zone (HZ) with the highest attack rates ≥30 per 100,000 inhabitants were considered to be the most affected by this infection according to DRC Health Ministry directives [23];
- Cluster analysis: Detection of spatial clusters of a disease is one of the most important techniques in epidemiology. For SaTScan (Software for the Space and Space-Time Scan Statistic) analysis, the location for health events can be points (representing addresses for patients) or centroids when considering geographic areas like township, region, district, health zone, area zone or village [27, 28]. In our study, a purely spatial scan analysis was carried out at HZ level to search for aggregates of cases for 2000–2012 (spatial clusters).

SaTScan is a spatial statistics Scan method for the detection and cluster inference in time-space under the relative risk (RR) hypothesis. The program applies a likelihood function to circular windows originating at defined locations of increasing size and compares observed and expected case numbers inside and outside the scan window to detect clusters that are least likely to have occurred by chance. The analyses were made using the discrete Poisson probability model (detection of clusters with values abnormally above the expected values of a binomial distribution) in a group of persons, a geographical entity or a lapse of time [29]. The geographic coordinates (latitude and longitude) were extracted from the DRC shapefile using the *R* software (see Additional file 2).

- Stationarity of meningitis cases: We tested the stationarity of meningitis cases in order to check whether the statistical properties of the national time-series process do not change over time [30]. For this, we first withdrawn from further analysis three outlier points with highest variance values corresponding to months 16, 23 and 38 (see Additional file 4). For the overall time-series of cases, we observed a very slight non-stationarity of mean values of meningitis cases per districts with time (*df* = 164, *z* = 2.311, *p* = 0.022), and a stationarity of variance values (*df* = 164, *z* = − 0.700, *p* = 0.485). According to these findings, we then decided to use time-series analyses as recommended for stationary data [30].
- Seasonal trend: The study of the temporal dynamics was first done in checking the existence of a seasonal variation across years drawing box-plot diagrams of total and mean cases per month. We also used the same procedure to visualize the mean and max attack rate values. Then we decomposed the total time-series and the time-series for the 3 clusters, i.e., primary cluster, secondary clusters 4 and 5, exhibiting more or less regular case notifications during 13 years (2000 to 2012) into a general tendency, a seasonal and residual components (see Additional file 3) according to Cleveland et al. (1990) using Cleveland’s algorithm in *R*. Autocorrelation in residual values was taken into account using autoregressive (AR), moving average (MA) or mixed (ARMA) methods [31, 32]. The use of the autocorrelation function (ACF) calculated on the residuals of the autoregressive model of order 1 was sufficient to obtain non-self-correlated residual values.

## Results

From 2000 to 2012, 92,492 cases including 11,630 deaths were reported in the 515 health zones (Case-fatality rate: 12.57%). The annual average attack rate of meningitis case notifications from the routine surveillance per 100,000 inh. is 10.67 (S.D ± 2.78). The highest lethal rate was observed in the former province of Bandundu (19.02%) and the lowest in the former Province Orientale (10.29%). The highest attack rates are observed in the provinces of Maniema (19.61 per 100,000 inh.), Kinshasa (18.72 per 100,000 inh.), in the former Orientale province (15.41 per 100,000 inh.), in the former Katanga (11.14 per 100,000 inh.), and in the former Kasaï Oriental province (9.60 per 100,000 inh.) (Table 1).

**Table 1:** Description of meningitis surveillance data in DRC, 2000–2012. (Source: Passive surveillance data, DLM, DRC)

N°	Provinces	Cases	Lethality (%)	Average annual attack rate (p. 100,000 inh.)	Standard Deviation (±)
1	Bandundu	7401	19.02	8.40	3.94
2	Bas Congo	1694	12.81	5.05	5.13
3	Equateur	9261	12.50	8.97	7.77
4	Kasaï Occidental	7105	13.89	9.31	5.44
5	Kasaï Oriental	9840	14.04	9.60	3.16
6	Katanga	14,051	10.31	11.14	4.60
7	Kinshasa	13,819	11.92	18.72	10.64
8	Maniema	4497	15.65	19.61	10.40
9	Nord Kivu	3682	12.68	5.38	2.45
10	Province Orientale	17,732	10.29	15.41	9.80
11	Sud Kivu	3410	11.35	6.08	2.69
	Total	92,492	12.57	10.67	2.78

### Laboratory confirmation

Between 2008 and 2012, 1.15% of total cases (486/42,256) had a lumbar puncture (LP) and 100% of these samples were tested through different lab methods (486/486) of which 54 positive (11%). All of 486 samples were tested by culture, 42.18% (205/486 samples of CSF) by soluble antigens search (latex agglutination) and 54.93% (267/486 samples of CSF) by gene amplification (Polymerase chain reaction).

The case confirmation database acquired at the INRB which includes the age and sex variables, the result of which is 119 female and 108 male for ≤12 months and 93 female and 135 male for > 12 months. If we consider only sex, we have 224 female and 262 male cases.

It’s important to note that the case confirmation database does not specify the age of 31 out of 486 patients whose CSF was tested for the period 2008 to 2012 (there are some accompanying children or mothers who did not give their age to health staff). A general description is given in Table 2.

**Table 2:** Confirmation of meningitis cases, DRC, 2008–2012

Serogroups/ Serotypes	Number	Percentage	Provinces
Collected samples	486		
Received samples	486	100	
Positive	54	11	
*Streptococcus pneumoniae*	27	50	Kinshasa, Katanga
*Haemophilus influenzae*	1	2	
Hi b	0	0	
Hi a	0	0	
Hi not serotyped	1	100	Katanga
*Neisseria meningitidis*	7	13	Province Orientale, Bandundu, Kinshasa
Nm A	3	43	Province Orientale
Nm B	0	0	
Nm C	4	57	Bandundu, Kinshasa
Nm W	0	0	
Other pathogens*	19	35	Kinshasa, Katanga and Bas-Congo
*Contaminated samples*	33	7	
*Sterile samples*	170	35	
*Negative samples*	229	47	

Note: Bacterial meningitis is mainly due to *Neisseria meningitidis* (Nm), *Streptococcus pneumonia*e (Sp) and *Haemophilus influenzae type b* (Hib). *Other pathogens causing meningitis include *Streptococcus* spp. (*n* = 1), *Streptococcus* group D (*n* = 5), *Salmonella* spp. (n = 1), *Enterobacter* spp. (*n* = 2), *Citrobacter* spp. (*n* = 3)*, Staphylococcus aureus* (n = 1), *Escherichia coli* (n = 1) and *Pseudomonas aeruginosa* (n = 1). (Source: INRB)

With a positivity rate of 11% (54/486), patients were primarily infected by *S. pneumoniae* (50%), *N. meningitidis* (13%), *H. influenzae* (2%) or other pathogens (35%) identified by CSF culture or PCRs performed. 486 CSF samples were tested via culture (100%) and 267 via PCR (54.93%).

### Spatial dynamics (2000 to 2012)

**Fig. 1:**
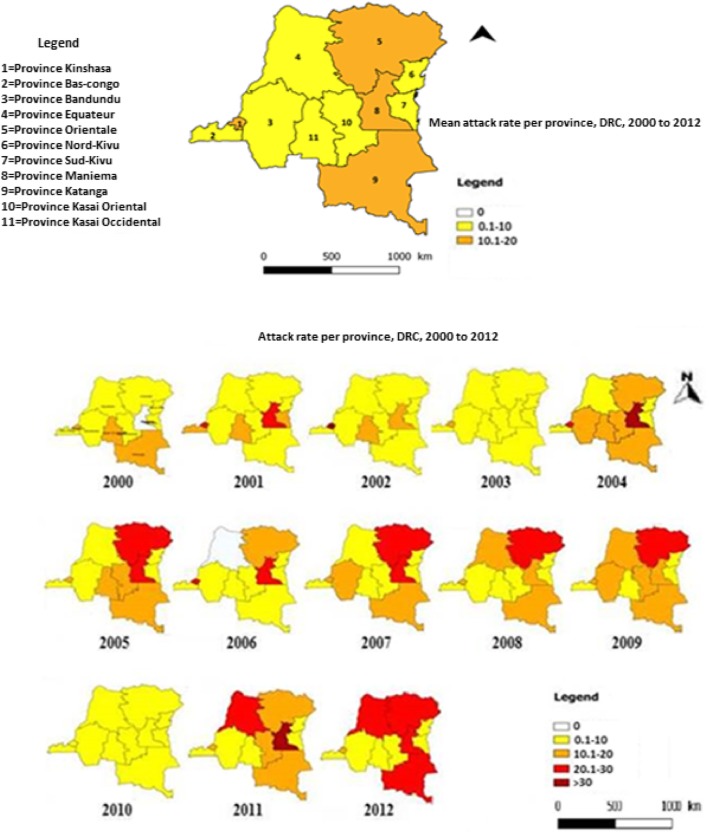
Evolution of the annual attack rate (per 100,000 inh.) of suspected cases of meningitis by province, DRC, 2000–2012. The map of the top represents the mean attack rate over the study period. (Source: The map was created with the provincial Shapefile obtained from Ministry of Health of DRC). The map was created using the free software QGIS® 1.8.0 geographical information system)

**Fig. 2:**
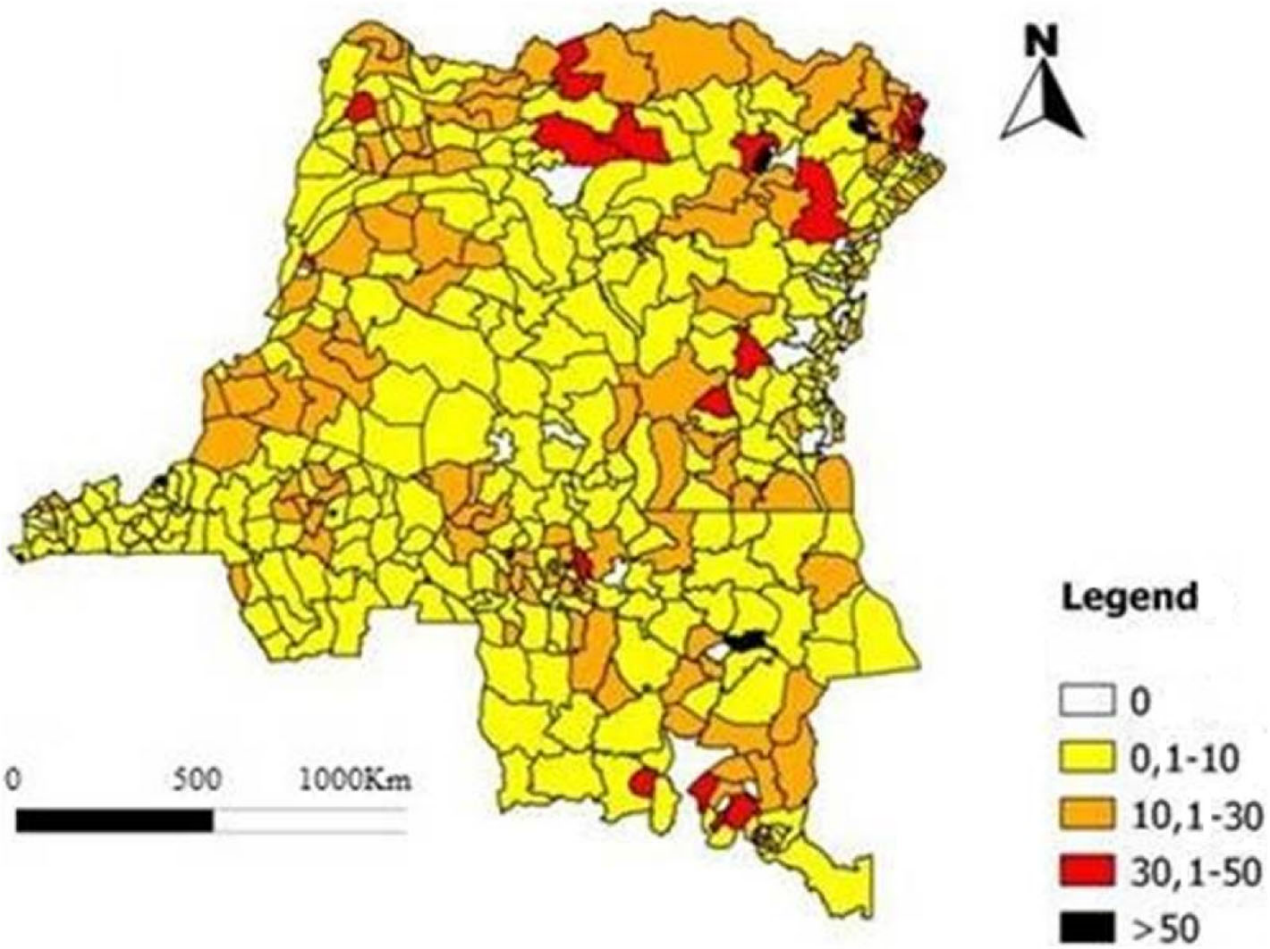
Average attack Rate (per 100,000 inh.) by Health Zone, DRC, 2000–2012. (Source: The map was created with the Health zones Shapefile obtained from Ministry of Health of DRC). The map was created using the free software QGIS® 1.8.0 geographical information system)

**Fig. 3:**
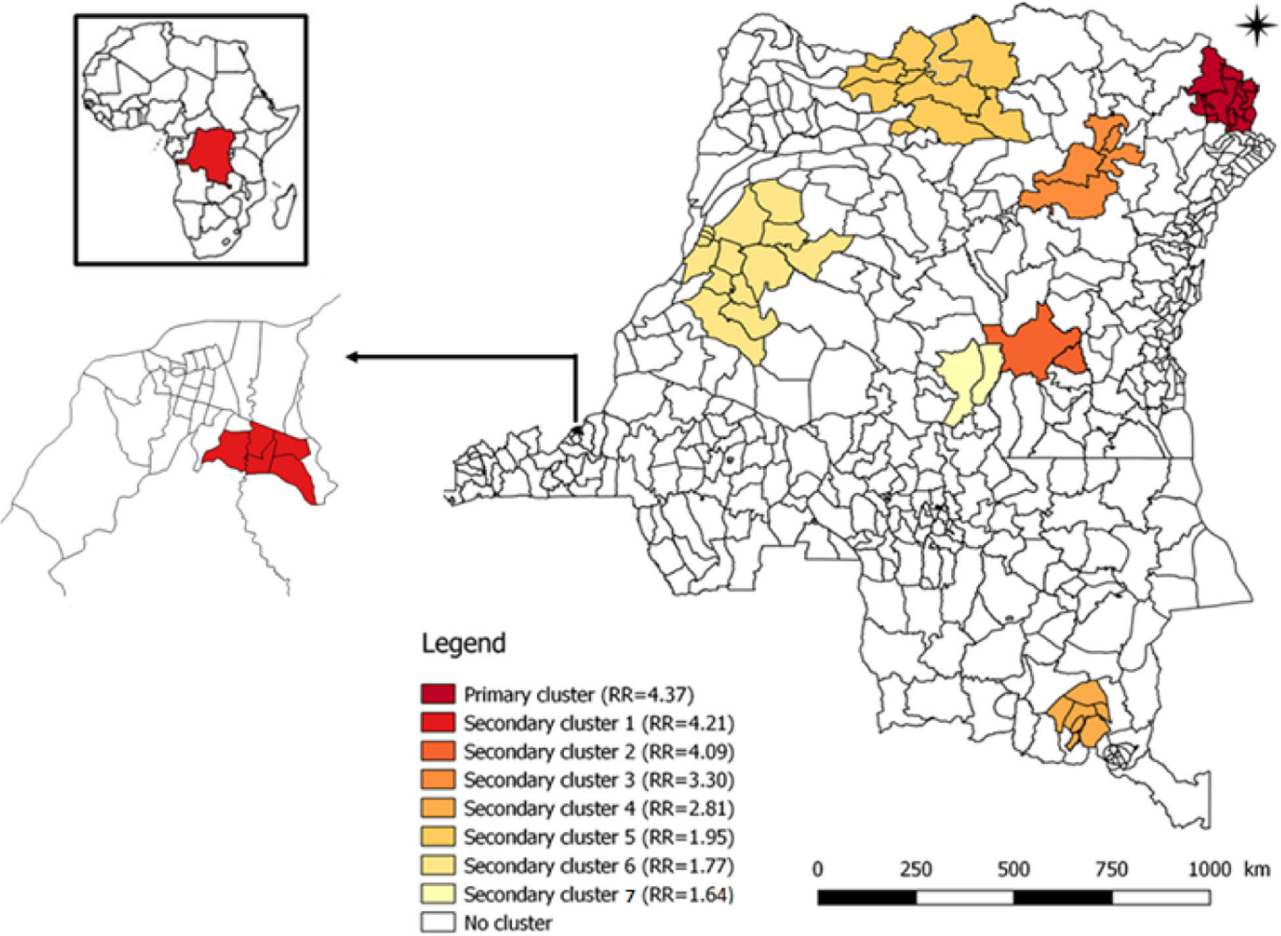
Spatial clusters associated to the risk of meningitis in 515 health zones, DRC, 2000–2012. (Source: The map was created with the Healh zones Shapefile obtained from Ministry of Health of DRC). The map was created using the free software SaTScan® v9.1.1

The spatial distribution of suspected meningitis cases revealed a strong spatial heterogeneity both at the provincial level and in the different health zones (see Figs. 1 and 2). The spatial analysis of meningitis cases at the scale of the 515 HZ identified 8 distinct clusters for meningitis, the most important of which being located in the former Orientale province and Kinshasa province. The former Orientale province is represented by 3 main clusters of which the most important (primary cluster) is represented by the HZ of Faradje, Makoro, Watsa, Laybo, Aba, Aungba, Ariwara, Adia, Adi, Aru and Biringi (Relative Risk = 4.37, *p*-value < 0.0001). Interestingly this primary meningitis cluster corresponds to the area where Ebola epidemic is currently occurring (at the date of 12th december 2019); the second is secondary cluster 3 formed by Bafwabogbo, Bafwasende, Wamba, Pawa and Isiro HZ (Relative Risk = 3.30, *p*-value < 0.0001); and the third is the part of secondary cluster 5 (Relative Risk = 1.90, *p*-value < 0.0001). A secondary cluster 1 in the Kinshasa province is constituted by Kingasani, Kimbanseke, Ndjili, Kisenso and Biyela HZ (Relative Risk = 4.21, *p*-value < 0.0001) (see Fig. 3 and Table 3).

**Table 3:** Spatial clusters associated to the risk of meningitis in 515 health zones, DRC, 2000–2012

N° Clusters	Relative Risk	Observed/expected cases	*p*-value	Provinces	Number of Health Zones	Health Zones
Primary cluster	4.37	4.11	< 0.0001	Orientale	11	Faradje, Makoro, Watsa, Laybo, Aba, Aungba, Ariwara, Adia, Adi, Aru and Biringi
Secondary cluster 1	4.21	4.00	< 0.0001	Kinshasa	6	Kingasani, Kimbanseke, Ndjili, Kisenso and Biyela
Secondary cluster 2	4.09	4.02	< 0.0001	Maniema	4	Kailo, Kinda, Alunguli and Kalima
Secondary cluster 3	3.30	3.25	< 0.0001	Orientale	5	Bafwabogbo, Bafwasende, Wamba, Pawa and Isiro
Secondary cluster 4	2.81	2.77	< 0.0001	Katanga	7	Kambove, Likasi, fungurume, Bunkeya, Kapolobwe, Kikula and Panda
Secondary cluster 5	1.90		< 0.0001	Equateur	5	Yakoma, Abuzi, Wapinda, Wasolo and Yamongili
				Orientale	5	Bondo, Likati, Bili, Monga and Aketi
Secondary cluster 6	1.77	1.77	< 0.0001	Kasaï Oriental	2	Djalo-Djeka and Katoko-Kombe
Secondary cluster 7	1.64	1.61	< 0.0001	Equateur	11	Lotumbe, Ingende, Iboko, Boende, Bikoro, Bolonge, Wangata, Monika, Bolomba, Mbandaka and Basankusu
				Bandundu	3	Penzwa, Kiri and Inongo

Clusters are distributed in provinces with high average attack rates, notably in the former Orientale province (average attack rates = 15.41 per 100,000 inh., *p* < 0.0001), which overlaps on 3 clusters (primary, secondary 3 and 5), in Kinshasa (average attack rate = 18.72 per 100,000 inh., *p* < 0.0001), in Maniema province (average attack rate = 19.61 per 100,000 inh., *p* < 0.0001), in the former province of Kasaï Oriental (average attack rate = 9.6 per 100,000 inh., *p* < 0.0001) and in the former Katanga (average attack rate = 11.14 per inh., *p* < 0.0001) (see Table 1).

### Seasonal and medium-time trends

**Fig. 4:**
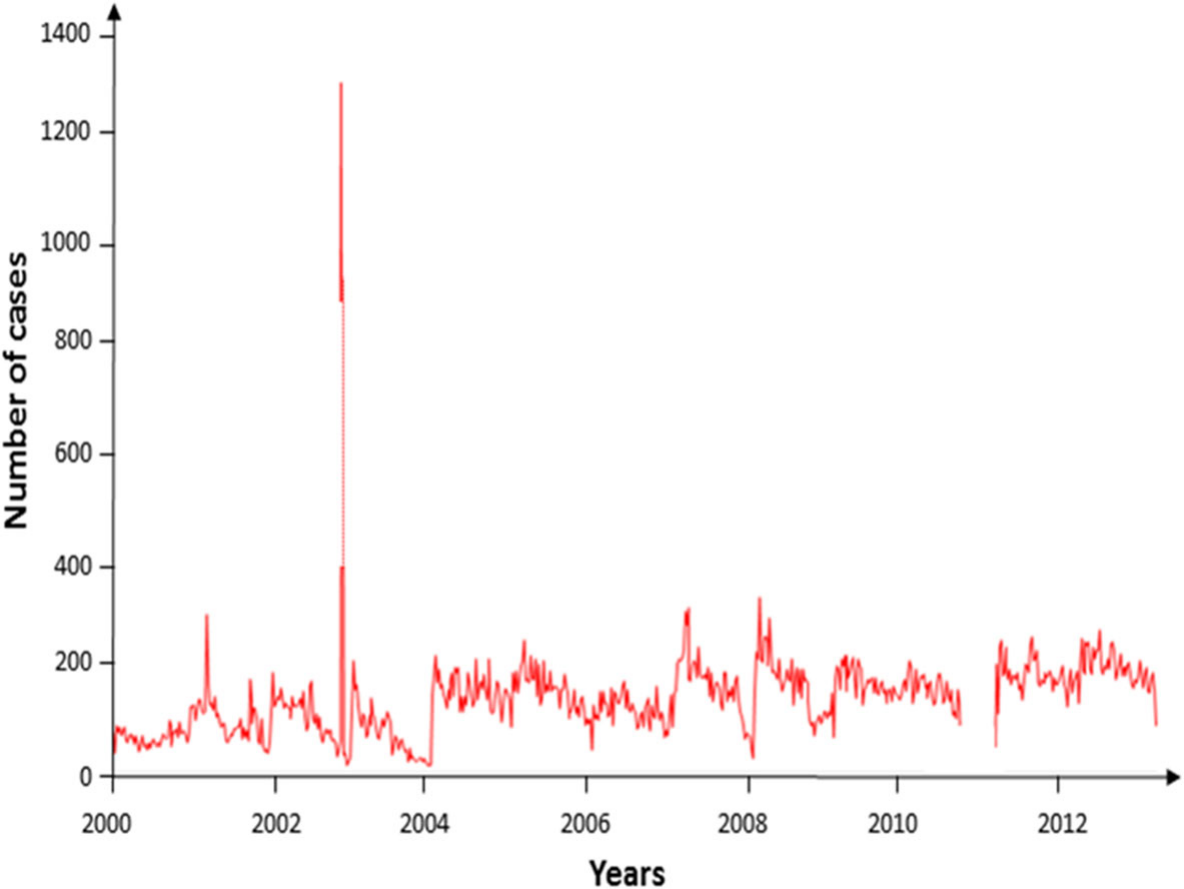
Weekly time-series of suspected meningitis cases, DRC, 2000–2012. Source: The graphic was created using the free software *R*® 3.0.1

At the national level, since 2000, there has been a steady trend in the number of cases over time to 2012 (Fig. 4). Concentrating on meningitis cases in the 8 clusters, the analysis of the periods of occurrence of major epidemics throughout the duration of the study and in the reports of investigations shows the concentration of these episodes during the short dry season in south and north Tropical Zones [from the beginning of February to fall March, or epidemiological week 5th (W5) to week 14 ^th^ (W14). Thus, epidemic peaks are mainly observed in 2001 (W7 to W12), in 2005 (W6 to W13), in 2007 (W5 to W10), in 2008 (W4 to W12) and in 2012 (W4 to W9) (see Additional file 5, Figure S 1). Low seasonal fluctuations of meningitis cases are observed when plotting both total and mean cases during years, with higher cases number observed from October to March and lower cases number from April to June (see Additional file 6, Figure S 2). The same results are obtained for mean and max attack rate values (see Additional file 6, Figure S 2).

Seasonal trends are sought by decomposing the time-series resulting from the clustering of geographically close and contiguous HZ by clusters. This decomposition in the time-series was done in the clusters where there is a regularity of case reporting of at least 4 years. Outbreaks were observed during the little dry season (W4 to W12) in all clusters but we have also some peaks in the rainy season (W40 to W48) (see Additional file 7, Figure S 3).

At primary cluster, an outbreak is observed from W7 to W44 in 2007 with a peak of 128 cases at W9 corresponding to February (dry season in this region), and the trend is increasing from 2004 (see Additional file 5, Figure S 1). However, seasonality explains a low proportion of the variability in disease cases with an order of 5% (6/120, 6 is the absolute average value of seasonality and 120 is the highest number of cases) (see Additional file 7, Figure S 3).

For secondary cluster 4, a case outbreak is observed from W1 to W17 with a peak of 68 cases at W4 in 2004 (little dry season in the southeast of the country) and another peak from W13 to W31 with an acme of 23 cases at W13 (small and large dry season in the southeast). The general trend of meningitis cases in time tends to show fluctuations since 2004. Seasonality explains in this cluster 12% of variation (3/25, 3 is the absolute average value of seasonality and 25 is the highest number of cases) (see Additional file 7, Figure S 3).

For secondary cluster 5, located in the northwest of DRC, an epidemic peak is observed from W1 to W18 in 2003 with a peak of 78 cases at W2 corresponding to February (dry season in this region). The trend is increasing since 2003 while seasonality explains only 4% in the variability of cases with time (3/80, three is the absolute average value of seasonality and 80 is the highest number of cases) (see Additional file 7, Figure S 3).

The decomposition of time-series for clusters reveals a low seasonal trend favoring the dry season for the development and spread of meningitis cases.

## Discussion

Known as a disease of the Sahelo-Sudanian region, punctuated by the breath of Harmattan winds [2, 19, 33, 34], meningitis cases reported outside the African meningitis belt raises the problem of knowledge on the epidemiology of epidemic meningitis in these non-endemic areas.

In the DRC, considering its current spatial distribution and mortality rate, bacterial meningitis is a public health problem in many different provinces.

At the health zone scale, the cluster analyses have revealed the existence of 8 clusters to be at higher risk for the development of meningitis outbreaks. These clusters are distributed in provinces with high average attack rates, notably in the former Orientale province, which overlaps on 3 clusters (primary, secondary 4 and 5) where the main strain was *Neisseria meningitidis* A, in Kinshasa where the main strains were *S. pneumoniae, N. meningitidis* A and C, in Maniema province, in the former province of Kasaï Oriental and in the former Katanga where the main strains were *S. pneumoniae*, *H. influenzae* and a secondary cluster 7 consisting by 11 HZ and 3 HZ respectively of former Equateur and Bandundu provinces where the main strain was *N. meningitidis* C. It is now crucial to try to better understand the environmental, ecological and population parameters that can explain the spatial diversity in the past and current meningitis distribution as observed in DRC. Environmental parameters including humidity, dusts and solid particles are likely to play a role along with population parameters including population movements due to insecurity in the northeast part of the country, and the difficulty to access to health centers for some human communities in the DRC [10, 22]. The next step is to quantify these parameters to explore their links with meningitis epidemics and patterns.

Regarding the laboratory data, 486 CSF were collected during the period of 2008–2012 which represents a low proportion of suspected cases, due to the weaknesses of the meningitis surveillance system in the DRC. On top of that, among these samples, only 11% (54/486) were positive. Indeed, 7% were contaminated, 35% were sterile and 47% negative samples (Table 2). These failures in diagnosis highlight the difficulties encountered by field health workers in DRC to perform disease samples and in good conditions of safety and transport [23, 35].

Additionally, the lethality observed (12.57%) is close to those observed in the countries of the meningitis belt (10 to 14%) [33], arguing again for the strong necessity to improve the laboratory diagnosis, strengthen surveillance and treatment of patients in DRC. In this country the diagnosis is mainly based on clinical definition of cases for surveillance.

Surveillance data for meningitis in the DRC have some limitations that should not bias our analyses. This is confirmed by a recent publication on the evaluation of IDSR in Africa with more than 85% data completeness and implementation of IDSR in 50 to 85% of structures at the peripheral level in 2017 [34]. First, these data correspond to suspected cases and a low proportion is actually tested for biological confirmation. Similarly to the studies performed in the African meningitis belt, the surveillance data do not reflect the exact number of meningitis cases with potential over estimations of cases, mainly during epidemics. Meanwhile, under-reporting of cases can occur because of the poor training of care providers and sometimes due to a lack of data transmission tools to the hierarchy. Biological confirmations of routine suspects cases are almost non-existent especially in rural areas. The biological confirmation protocol of the cases defined by the WHO requires a respect of norms and a technical platform not always accessible in the structures of care (reagents, materials for cerebrospinal fluid transport, culture and others). Difficulties related to the collection of cerebrospinal fluid (CSF) are transport and the use of certain methods of laboratory diagnosis (e.g., microscopic exam, culture, biochemical analyses useful for diagnosis, soluble antigens search, Gene amplification ...).

In DRC, data quality control is performed internally within the Directorate for Disease Control at a quarterly pace. And this direction is also assessed by the DRC General Secretariat for Health at a quarterly rate and during these evaluations; special attention is paid to the quality of surveillance data. Also, annually, the Directorate for Disease Control in particular and the Ministry of Health in general are subject to external quality control of surveillance data (by South Africa national laboratory and CDC Atlanta for Laboratory Data).

Another limitation is the population data, which correspond to estimates based on a 1984 census with application of a calculated annual growth rate of 1.03%. That is the only one available and accepted by all actors in this country.

The biological confirmation data are useful for determining the case confirmation rate that remains low for several reasons, including the low availability of diagnostic materials and inputs in provinces, but also the transport of CSF samples (transport medium Trans-isolate from cerebrospinal fluid). Despite the low numbers of confirmed cases, we observed diversity in pathogens responsible (*N. meningitidis*, *S. pneumoniae*, *H. influenzae* and other pathogens) for causing meningitis in DRC.

Since 2015, MenAfricVac vaccine which contributes to the decline in the incidence of suspected meningitis cases in Africa [36, 37], and which only targets MenA, has been implemented in DRC in former province Orientale, Nord Kivu and Sud Kivu provinces with high incidence, while MenC (against which the vaccine is not efficient) is circulating in other provinces, e.g., former Bandundu Province and Kinshasa. This issue deserves further exploration in order to adapt the vaccination strategy with the current epidemiological situation by notably using polyvalent vaccines especially in the routine vaccination. It should be noted that the PCV-13 vaccine has been introduced in the DRC since January 2011 [38].

In the African meningitis belt, it is well known now that the influence of Harmattan winds and dust during the dry season can explain the seasonality of the disease [10, 19, 39–41]. In areas outside the belt, meningitis epidemics may occur in tropical forest-type conditions as for DRC, and sometimes in very isolated and sparsely populated areas such as the one that occurred in the Inongo area in the former Bandundu province in 2002 [42].

From our present study, low defined seasonality of meningitis was observed with higher number of cases mainly observed during the dry season, i.e., from October to March. Even if these results are similar to those of other studies conducted in the countries of the meningitis belt [3, 4, 20, 43], the low seasonal trend that we observed in our study is new. However, we observed an important heterogeneity in seasonal variation across different years and different health zones of DRC. Indeed a low seasonal for 3 clusters reveals trend favoring the dry season for the development and spread of meningitis cases although it is not the only factor associated with seasonal outbreaks of meningitis. Some disease peaks may also occur during the rainy season.

For instance, global patterns of influenza seasonal activity have shown that influenza virus dynamics displays strong seasonal cycles in temperate areas of the world and less defined seasonality in tropical regions, suggesting that bioclimatic factors may drive these seasonal disease patterns [44, 45]. Our observation for meningitis dynamics would tend to indicate the existence of the same global patterns, with less defined seasonality for meningitis in regions close to the Equator [44, 45]. However, disease data are still scarce in many African countries in order to more critically test proposed mechanisms responsible for the amplitude and timing of seasonal variation in meningitis cases.

Hence the hypothesis of a combination of factors including low humidity, increasing the amount of dust, freshness promoting irritation of the mucous membranes of the upper respiratory tract, promiscuity especially for children of school age (holiday season) and other parameters would promote human-to-human transmission. However, the strong observed residues in the decomposition into time-series of three clusters suggest that, apart from low seasonality, there are other factors that explain this temporal dynamic, which remains entirely to be explored in regions located outside the meningitis belt.

Several hypotheses can be proposed from the influence of climate change on ecosystems to the adaptation of pathogens to ecosystems that until then were probably not very favorable to the development and spread of meningitis in central Africa. An ecological/environmental hypothesis could also be discussed. Indeed, the continuous and accelerated deforested areas over the past half-century in the central basin of enlarged Africa to neighboring forest regions of southern Africa [46], would probably reveal through these epidemics one of these most unexpected consequences.

Deforestation could contribute to the creation of a corridor that can facilitate wind circulation and probably of the germs responsible for the disease. The global warming that is already manifesting itself in the ecosystems modification of Central and Southern Africa [47] could certainly be a factor of amplification and acceleration of these health issues. Migratory movements and increased population pressure could also contribute to the spread of the disease in DRC [48, 49]. Finally, there is a need to vaccinate the population against the different strains of pathogens found, achieving access to appropriate diagnostic tests at all levels of care, to enhance surveillance and ensure patients can be promptly treated through effective antibiotics and adjunctive care in this country in order to better control the disease.

## Conclusion

The present study has identified major meningitis clusters in the DRC, bringing together 8 health zones at higher risk of outbreaks of epidemics (attack rate varying between 10 and 50 per 100,000 inh.). Most of clustered areas for meningitis are located in the northeast and southeast regions of the country and in Kinshasa province located in the southwest.

These clusters provide the first localization of areas potentially at risk of meningitis development in DRC, and this could constitute a first approach for targeting the sentinel site surveillance strategies in this country. In addition, we show a slight seasonal variation in meningitis patterns, with the highest number of cases observed during the dry period, which is consistent with what is already observed for meningitis epidemics in the African meningitis belt. Nevertheless, the low seasonal variation observed in DRC compared to meningitis trend observed in Sahelo-Sudanian countries would tend to indicate that environmental, bioclimatic factors are less important in driving seasonal meningitis epidemic patterns, or that other factors may scramble this seasonal trend.

Subsequent work in health zones belonging to these clusters searching for environmental factors that may explain the occurrence of cases and epidemic patterns should be promoted in order to better understand the eco-epidemiology of this infectious disease in central Africa. The next step is now to explore the environmental, geographic, demographic and socio-anthropological parameters to explain the temporal and spatial distribution of meningitis cases in this region located outside the so-called meningitis belt. An enhanced surveillance is highly recommended and such spatial study can also provide the health authorities with insights that help to target high risk population in order to prevent the development of meningitis epidemics and other diseases like Ebola virus disease, measles and numerous other ones.

## Supplementary information

**Additional file 1.** Data cases and deaths from 2000 to 2012, allowing the calculation of annual attack rates by province and the average attack rate by health zone and by province and the standard deviation from 2000 to 2012.

**Additional file 2.** Case database, average population from 2000 to 2012 and geographic coordinates (latitude and longitude), which allowed the identification of primary and secondary clusters using Satscan software.

**Additional file 3.** The data file used to produce weekly time-series of meningitis suspected cases in the 8 clusters, DRC, 2000–2012 and Time-series decomposition using LOESS regression for meningitis of primary cluster, secondary cluster 4 and secondary cluster 5.

**Additional file 4.** File of average and variance of the cases in the Health zones for the data stationarity’s study. (XLS 46 kb)

**Additional file 5: Figure S1.** Weekly time-series of meningitis suspected cases in the 8 clusters corresponding to Fig. 3, DRC, 2000–2012.

**Additional file 6: Figure S2.** Box-plot illustrations showing for 1) upper panel, left mean cases of meningitis, right total cases of meningitis, and 2) lower panel, left max attack rate, and right mean attack rate, the variations during years. The red curves show the locally weighted non parametric regressions using a LOESS function (tension, *t* = 0.5), DRC, 2000–2012.

**Additional file 7: Figure S3.** Time-series decomposition using LOESS regression for meningitis, for primary cluster, secondary cluster 4 and secondary cluster 5, DRC, 2000–2012.

## Data Availability

All data generated or analysed during this study are included in this published article [and its supplementary information files].

## Abbreviations

ACF: Autocorrelation function
AR: Autoregressive
ARMA: Autoregressive moving average
CSF: Cerebrospinal fluid
*df*: Degree of freedom
DRC: Democratic Republic of Congo
EPI: Expanded Programme on Immunization
Hia: *Haemophilus influenzae* type a
Hib: *Haemophilus influenzae* type b
HZ: Health zone
IDSR: Integrated Diseases Surveillance System
INRB: Institut National de Recherche Biomédicale
MA: Moving average
Nm: *Neisseria meningitidis*
*p*: *p*-value
PCR: Chain Reaction
PEV: Programme Elargi de vaccination
S.D.: Standard deviation
Sp: *Streptococcus pneumoniae*
W: Week
WHO: World Health Organization
