# Supplementary material for: Understanding the spatio-temporal dynamics of meningitis epidemics outside the belt: the case of the Democratic Republic of Congo (DRC)
Source: BMC Infect Dis. 2020 Apr 20;20:291. doi: 10.1186/s12879-020-04996-7 (PMC7168871; doi:10.1186/s12879-020-04996-7)
Supplement: Supplementary file 5 — Additional file 5: Figure S1. Weekly time-series of meningitis suspected cases in the 8 clusters corresponding to Fig. 3, DRC, 2000–2012. [file 12879_2020_4996_MOESM5_ESM.doc]

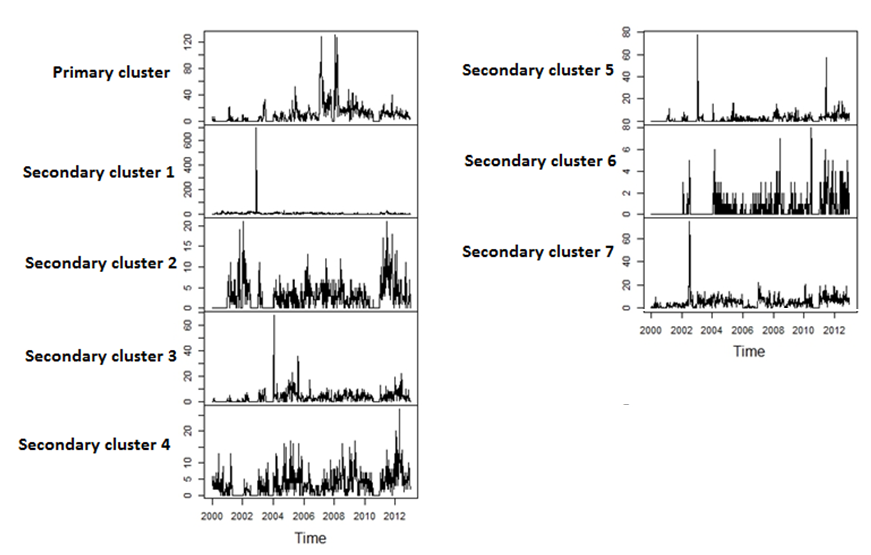


Additional file 5 (Figure S1): Weekly time-series of meningitis suspected cases in the 8 clusters corresponding to Figure 3, DRC, 2000-2012.

Source: The graphics were created using the free software *R*® 3.0.1.
