# Supplementary material for: Understanding the spatio-temporal dynamics of meningitis epidemics outside the belt: the case of the Democratic Republic of Congo (DRC)
Source: BMC Infect Dis. 2020 Apr 20;20:291. doi: 10.1186/s12879-020-04996-7 (PMC7168871; doi:10.1186/s12879-020-04996-7)
Supplement: Supplementary file 6 — Additional file 6: Figure S2. Box-plot illustrations showing for 1) upper panel, left mean cases of meningitis, right total cases of meningitis, and 2) lower panel, left max attack rate, and right mean attack rate, the variations during years. The red curves show the locally weighted non parametric regressions using a LOESS function (tension, t = 0.5), DRC, 2000–2012. [file 12879_2020_4996_MOESM6_ESM.doc]

**
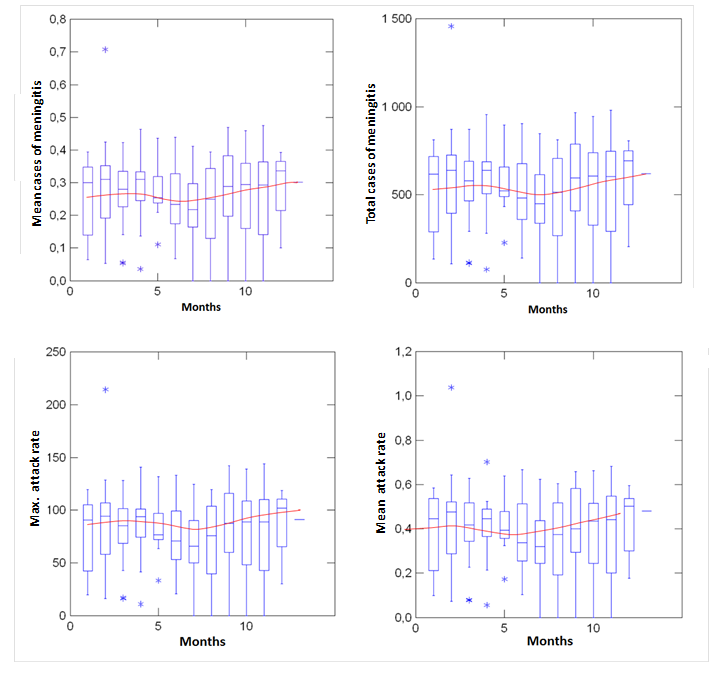
**

Additional file 6 (Figure S 2): Box-plot illustrations showing for upper panel, left mean cases of meningitis, right total cases of meningitis, lower panel, left max attack rate, and right mean attack rate, the variations during years. The red curves show the locally weighted non parametric regressions using a LOESS function (tension, *t*=0.5), DRC, 2000-2012.

Source: The graphics were created using the software Stata® ver. 16.
