# Supplementary material for: Understanding the spatio-temporal dynamics of meningitis epidemics outside the belt: the case of the Democratic Republic of Congo (DRC)
Source: BMC Infect Dis. 2020 Apr 20;20:291. doi: 10.1186/s12879-020-04996-7 (PMC7168871; doi:10.1186/s12879-020-04996-7)
Supplement: Supplementary file 7 — Additional file 7: Figure S3. Time-series decomposition using LOESS regression for meningitis, for primary cluster, secondary cluster 4 and secondary cluster 5, DRC, 2000–2012. [file 12879_2020_4996_MOESM7_ESM.doc]

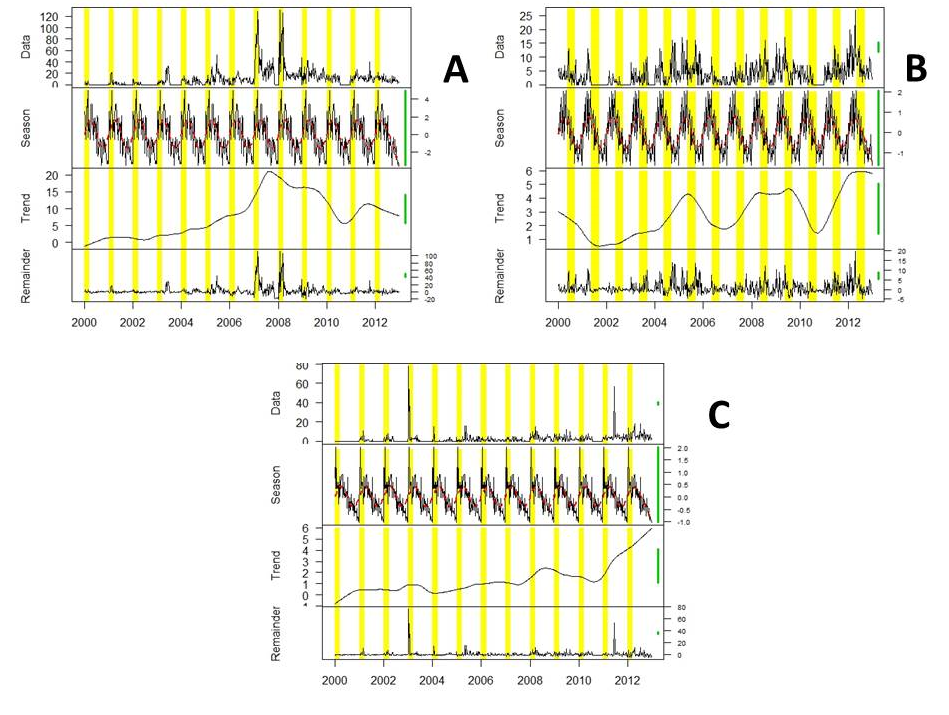


**Additional file 7 (Figure S 3): (A)** Time-series decomposition using LOESS regression for meningitis, for the primary cluster, DRC, 2000-2012. Meningitis slightly varies seasonally. The general trend of meningitis cases is increasing from 2004 to 2007-2008, and then is dropping down. Remainder (residuals) explained 83 % of model variance, trend 15% and seasonality 5% only. Green line indicates average values for seasonality, trend and residuals axes. **(B)** Time-series decomposition using LOESS regression for meningitis, for the secondary cluster 4, DRC, 2000–2012. Meningitis slightly varies seasonally. The general trend of meningitis cases shows the existence of waves on a medium-time scale since 2003-2004. Remainder (residuals) explained 80 % of model variance, trend 5% and seasonality 12 % only. Green line indicates average values for seasonality, trend and residuals axes**.** **(C)** and Time-series decomposition using LOESS regression for meningitis, for the secondary cluster 5, DRC, 2000–2012. Meningitis slightly varies seasonally. The general trend shows an increase of meningitis cases since 2003. Remainder (residuals) explained 80% of model variance, trend 4% and seasonality 4% only. Green line indicates average values for seasonality, trend and residuals axes. Disease case trends for these three clusters indicate a non-stationarity, which for the present work was not explored further and would merit a more powerful statistical analysis.

Source: The graphics were created using the free software *R*® 3.0.1.
